# Statin use and survival in patients with metastatic castration-resistant prostate cancer treated with abiraterone or enzalutamide after docetaxel failure: the international retrospective observational STABEN study

**DOI:** 10.18632/oncotarget.24888

**Published:** 2018-04-13

**Authors:** Jacob A. Gordon, Carlo Buonerba, Gregory Pond, Daniel Crona, Silke Gillessen, Giuseppe Lucarelli, Sabrina Rossetti, Tanya Dorff, Salvatore Artale, Jennifer A. Locke, Davide Bosso, Matthew Ivan Milowsky, Mira Sofie Witek, Michele Battaglia, Sandro Pignata, Cyrus Cherhroudi, Michael E. Cox, Pietro De Placido, Dario Ribera, Aurelius Omlin, Gaetano Buonocore, Kim Chi, Christian Kollmannsberger, Daniel Khalaf, Gaetano Facchini, Guru Sonpavde, Sabino De Placido, Bernhard J. Eigl, Giuseppe Di Lorenzo

**Affiliations:** ^1^ Vancouver Prostate Center, Vancouver Coastal Health Research Institute, Vancouver, British Columbia, Canada; ^2^ Department of Clinical Medicine and Surgery, University of Naples Federico II, Naples, Italy; ^3^ Istituto Zooprofilattico Sperimentale del Mezzogiorno, Portici, Italy; ^4^ McMaster University, Hamilton, Ontario, Canada; ^5^ Lineberger Comprehensive Cancer Center, University of North Carolina, Chapel Hill, North Carolina, USA; ^6^ Department of Medical Oncology, Cantonal Hospital St. Gallen, St. Gallen, Switzerland; ^7^ Department of Emergency and Organ Transplantation, Urology, Andrology and Kidney Transplantation Unit, University of Bari, Bari, Italy; ^8^ S.S.D Oncologia Clinica Sperimentale Uro-Andrologica, Dipartimento Corp-S Assistenziale dei Percorsi Oncologici Uro-Genitale, Istituto Nazionale Tumori Fondazione G. Pascale-IRCCS, Naples, Italy; ^9^ University of Southern California Keck School of Medicine, Norris Comprehensive Cancer Center, Los Angeles, California, USA; ^10^ Oncology Department, Ospedale di Gallarate ASST Valle Olona, Gallarate, Italy; ^11^ Division of Medical Oncology, Department of Uro-Gynecologi cal Oncology, Istituto Nazionale Tumori Fondazione G. Pascale-IRCCS, Naples, Italy; ^12^ Faculty of Medicine, University of Ottawa, Ottawa, Ontario, Canada; ^13^ Hospital Directorate, Azienda Ospedaliera Universitaria Federico II of Naples, Naples, Italy; ^14^ BC Cancer, Vancouver, British Columbia, Canada; ^15^ Genitourinary Oncology Section, Dana Farber Cancer Institute, Boston, Massachusetts, USA

**Keywords:** prostate cancer, abiraterone, enzalutamide, statins

## Abstract

**Background:**

Statins may potentiate the effects of anti-hormonal agents for metastatic castration-resistant prostate cancer (mCRPC) through further disruption of essential steroidogenic processes. We investigated the effects of statin use on clinical outcomes in patients with mCRPC receiving abiraterone or enzalutamide.

**Materials and methods:**

This was a retrospective multicenter study including patients that received abiraterone or enzalutamide for mCRPC. The effect of concurrent statin use on outcomes was evaluated. The associations of statins with early (≤12 weeks) prostate-specific antigen (PSA) declines (> 30%), cancer-specific survival and overall survival (OS) were evaluated after controlling for known prognostic factors.

**Results:**

Five hundred and ninety-eight patients treated with second-line abiraterone or enzalutamide after docetaxel for mCRPC were included. A total of 199 men (33.3%) received statins during abiraterone/enzalutamide treatment. Median OS was 20.8 months (95% CI = 18.3–23.2) for patients who received statins, versus 12.9 months (95% CI = 11.4–14.6) for patients who did not receive statins (*P* < 0.001). After adjusting for age, alkaline phosphatase, PSA, neutrophil-to-lymphocytes ratio, Charlson comorbidity score, Gleason score, visceral disease, hemoglobin, opiate use and abiraterone versus enzalutamide treatment, the use of statin therapy was associated with a 53% reduction in the overall risk of death (hazard ratio [HR] = 0.47; 95% CI = 0.35–0.63; *P* < 0.001). Statin use was also associated with a 63% increased odds of a > 30% PSA decline within the first 12 weeks of treatment (OR = 1.63; 95% CI = 1.03–2.60; *P* = 0.039).

**Conclusions:**

In this retrospective cohort, statin use was significantly associated with both prolonged OS and cancer-specific survival and increased early > 30% PSA declines. Prospective validation is warranted.

## INTRODUCTION

In developed countries, prostate cancer is the most prevalent malignancy in men, with 142,000 patients dying each year, and an 8.8% cumulative life-time incidence [1]. Statins are a therapeutic class of medications that are commonly prescribed to lower circulating cholesterol levels through inhibition of 3-hydroxy-3-methylglutaryl coenzyme A (HMG-CoA) reductase [2], and have an established role in primary and secondary cardiovascular prevention [3]. Over the past decade, a preponderance of evidence from numerous studies, mostly conducted in patients with hormone-sensitive disease, has shown that statin use in prostate cancer patients is associated with longer cancer-specific and overall survival (OS) [4]. The putative mechanism for this observed improvement in survival is that statins may impair prostate cancer growth via multiple cholesterol- and non-cholesterol-mediated effects [4]. In a recently published study of a large, registry-based cohort, which included >30,000 prostate cancer patients [5], statin use was predictive of improved cancer-specific and OS, after adjusting for stage, Gleason score and primary treatment at diagnosis. Conversely, there is little evidence regarding the effects of statins among patients with castration-resistant prostate cancer (CRPC), and the potential synergism with active systemic treatments (e.g., abiraterone and enzalutamide).

Abiraterone works by inhibiting residual adrenal and intra-tumoral androgen synthesis via CYP17A blockade [6], while enzalutamide acts by inhibiting binding of testosterone to the androgen receptor (AR) as well as by blocking androgen-mediated change and nuclear translocation of AR [7]. In one small retrospective study, statin use was significantly associated with longer OS and early PSA declines in men who received abiraterone [8]. In contrast, this OS advantage has not been consistently observed in other studies [9, 10]. Furthermore, there is prospective evidence from a phase III trial suggesting that statins may be discontinued in the palliative care setting with no detrimental effect on survival [11].

In view of the potential additive effect of statins with novel hormonal agents and of the unknown value of continuing versus discontinuing statin therapy in patients with metastatic CRPC (mCRPC), a multi-center retrospective study was conducted to further explore the effects of statin use on PSA response and survival outcomes during second-line (post-docetaxel) treatment with abiraterone or enzalutamide, after adjusting for multiple known predictive factors in the second-line setting [12].

## RESULTS

### Patients’ characteristics and outcomes

Six hundred and forty-two patients were initially included in this dataset. Of these, 44 patients were excluded because statin use could not be ascertained. Baseline characteristics and outcomes are presented for the remaining 598 patients in Table 1A–1D. Notably, > 50% of patients came from one treatment center (BCCA) and an additional 21% of patients came from a second center (Federico II Napoli). Median age of the population was 72 years (range, 42–96). Most of the study patients received abiraterone. Median duration of second-line treatment with abiraterone or enzalutamide was 8.3 months (range, 0.4–47.5), with 52% of patients having a > 30% PSA decrease within the first 12 weeks of treatment. At the time of this analysis, 513 (85.8%) patients had died, with a median OS of 16.1 months (95% confidence interval [CI] = 13.8–17.0). Cancer-specific survival was 16.2 months (95% CI: 14.3–17.1).

**Table 1A:** Summary statistics

Characteristic	Statistic	*N*	All Patients	Abiraterone	Enzalutamide
**Site**	*Federico II Napoli* *Pascale Napoli* *University Bari* *St. Gallen* *UNC* *UCLA* *BCCA* *Gallarate*	598	127 (21.2) 17 (2.8) 21 (3.5) 29 (4.9) 41 (6.9) 15 (2.5) 342 (57.2) 6 (1.0)	91 (19.0) 14 (2.9) 13 (2.7) 27 (5.6) 20 (4.2) 9 (1.9) 301 (62.7) 5 (1.0)	36 (30.5) 3 (2.5) 8 (6.8) 2 (1.7) 21 (17.8) 6 (5.1) 41 (34.8) 1 (0.9)
**Age**	*Mean (std dev*) *Median (range)*	598	72.5 (9.0) 72 (42, 96)	72.6 (9.0) 72 (42, 96)	72.0 (8.8) 72 (43, 90)
**Gleason Score**	*N (%) ≥8*	540	306 (56.7)	248/431 (57.5)	58/109 (53.2)
**Charlson Score**	*Median (range*) *N (%) ≥10*	598	10 (6, 17) 341 (57.0)	10 (6, 17) 274/480 (57.1)	10 (6, 15) 67/118 (56.8)
**Baseline PSA**	*Median (range)*	588	87.3 (0, 7938)	97.8 (0, 7938)	61 (1.9, 2220)
**Alkaline Phosphatase**	*Median (range)*	448	119 (8.9, 2189)	120 (8.9, 2189)	105 (39, 1791)
**LDH**	*Median (range)*	259	264 (90, 2598)	262 (90, 2598)	266 (103, 2219)
**Neutrophils/Lymphocyte Ratio**	*Median (range)*	530	3.4 (0.2, 37.5)	3.5 (0.2, 34.5)	2.7 (1.0, 37.5)
**Hemoglobin**	*Median (range)*	555	11.9 (5.7, 15.8)	11.9 (5.7, 15.8)	11.8 (7.1, 15.6)
**Months, Castration-sensitive Disease**	*Median (range)*	390	18.4 (0.2, 65.5)	18.6 (0.2, 65.5)	16.0 (0.8, 59.8)
**Months, Diagnosis to Mets**	*Median (range)*	474	37.0 (0, 162.0)	39.3 (0, 161.3)	25.0 (0, 162.0)
**Opiate Use**	*N (%) Yes*	587	191/587 (32.5)	152/476 (31.9)	39/111 (35.1)
**Visceral Disease**	*N (%) Yes*	598	46 (7.7)	31/480 (6.5)	15/118 (12.7)
**Treatment with abiraterone/enzalutamide ± statins**					
**Treatment**	*N (%) Abiraterone*	598	480 (80.3)	480 (100.0)	0 (0.0)
**Concomitant Statins**	*N (%) Yes* *Atorvastatin* *Lovastatin* *Pravastatin* *Rosuvastatin* *Simvastatin* *Unknown*	598	199/598 (33.3) 107 (53.8) 3 (1.5) 11 (5.5) 33 (16.6) 22 (11.1) 23 (11.6)	157/480 (32.7) 93 (59.2) 2 (1.3) 8 (5.1) 30 (19.1) 20 (12.7) 4 (2.6)	42/118 (35.6) 14 (33.3) 1 (2.4) 3 (7.1) 3 (7.1) 2 (4.8) 19 (45.2)
**Dose of Statins**	*Median (range)*	122	20 (5, 80)	20 (5, 80)	20 (5, 40)
**Simvastatin Equivalent Dose**	*Median (range)*	122	30 (8, 120)	30 (8, 120)	30 (10, 60)
**Statins Prior to Abiraterone/Enzalutamide**	*N (%) Yes*	196	191/196 (97.5)	151/154 (98.1)	40/42 (95.2)
**Statin Use Suspended during abiraterone/enzalutamide treatment**	*N (%) Yes*	196	3/196 (1.5)	2/154 (1.3)	1/42 (2.4)
**Months, Duration of Abiraterone/Enzalutamide Treatment**	*Median (range)*	183	8.3 (0.4, 47.5)	8.5 (0.4, 47.5)	7.1 (1.4, 33.4)
**Use Hydrophilic Statin**	*N (%) Yes*	176	44 (25.0)	38/153 (24.8)	6/23 (26.1)
**Source of Statin Use Data**	*Prescription data* *Claims*	598	543 (90.8%) 55 (9.2%)	444 (92.5%) 36 (7.5%)	99 (83.8%) 19 (16.1%)

Characteristics of the study population grouped by treatment.

**Table 1B:** Outcomes of the study population, grouped by treatment

Characteristic	Statistic	*N*	All Patients	Abiraterone	Enzalutamide
Outcomes					
**>30% PSA Decline at Week 4**	*N (%) Yes*	519	209 (40.3)	169/419 (40.3)	40/100 (40.0)
**>30% PSA Decline at Week 8**	*N (%) Yes*	480	223 (46.5)	184/391 (47.1)	39/89 (43.8)
**>30% PSA Decline at Week 12**	*N (%) Yes*	469	231 (49.3)	184/383 (48.0)	47/86 (54.7)
**>30% PSA Decline at 4,8 or 12 Weeks†**	*N (%) Yes*	574	299/574 (52.1)	243/465 (52.3)	56/109 (51.4)
**Overall Survival**	*N (%) Deaths* *Median (95% CI)* *6-mo OS (95% CI)* *1-year OS (95% CI)* *2-year OS (95% CI)*	598	513 (85.8) 16.1 (13.8, 17.0) 81.7 (78.3, 84.6) 61.0 (56.9, 64.8) 31.2 (27.5, 35.1)	424 (88.3) 15.8 (13.7, 17.0) 82.4 (78.7, 85.6) 61.3 (56.7, 65.5) 30.5 (26.3, 34.7)	89 (75.4) 16.5 (12.1, 20.1) 78.5 (69.9, 84.9) 59.7 (50.1, 68.1) 34.7 (25.8, 43.7)
**Cause of Death**	*Prostate Cancer*	598	468 (91.2)	390/424 (92.0)	78/89 (87.6)
**Cancer-Specific Survival**	*Median (95% CI)* *6-mo OS (95% CI)* *1-year OS (95% CI)* *2-year OS (95% CI)*	598	16.5 (15.3, 17.7) 82.7 (79.4, 85.6) 63.2 (59.1, 67.0) 33.8 (29.8, 37.8)	16.4 (14.6, 17.7) 83.4 (79.7, 86.5) 63.3 (58.7, 67.5) 33.0 (28.6, 37.4)	17.6 (13.6, 21.4) 80.0 (71.4, 86.2) 63.2 (53.5, 71.4) 37.4 (28.0, 46.8)
**Vascular Events**	*Cardiovascular N (%)* *Cerebrovascular N (%)* *Either N (%)*	598	20 (3.3) 13 (2.2) 33 (5.5)	15 (3.1) 12 (2.5) 27 (5.6)	5 (4.2) 1 (0.9) 6 (5.1)

^†^denominator is number of patients with a PSA assessment at week 4, 8 or 12.

**Table 1C:** Summary statistics

Characteristic	Statistic	N	No Statins	N	Statins
**Site**	*Federico II of Napoli* *Pascale Napoli* *University of Bari* *St. Gallen* *UNC* *UCLA* *BCCA* *Gallarate*	399	74 (18.6) 8 (2.0) 14 (3.5) 25 (6.3) 27 (6.8) 7 (1.8) 241 (60.4) 3 (0.8)	199	53 (26.6) 9 (4.5) 7 (3.5) 4 (2.0) 14 (7.0) 8 (4.0) 101 (50.8) 3 (1.5)
**Age**	*Mean (std dev*) *Median (range)*	399	71.9 (9.4) 72 (42, 96)	199	73.8 (7.9) 74 (43, 94)
**Gleason Score**	*N (%) ≥8*	354	204 (57.6)	186	102 (54.8)
**Charlson Score**	*Median (range*) *N (%) ≥10*	399	10 (6, 15) 206 (51.6)	199	10 (6, 17) 135 (67.8)
**PSA at Diagnosis**	*Median (range)*	391	95.3 (0, 7149)	197	80 (0.2, 7938)
**Alkaline Phosphatase**	*Median (range)*	312	113 (8.9, 2189)	136	120 (25, 1791)
**LDH**	*Median (range)*	175	260 (103, 2598)	136	272 (90, 2219)
**Neutrophils/Lymphocyte Ratio**	*Median (range)*	358	3.4 (0.2, 34.5)	172	3.3 (0.2, 37.5)
**Hemoglobin**	*Median (range)*	373	11.9 (5.7, 15.8)	182	12.0 (7.9, 15.5)
**Months, Castration-sensitive Disease**	*Median (range)*	259	18.4 (0.2, 65.5)	131	18.4 (0.6, 65.4)
**Months, Diagnosis to Metastases**	*Median (range)*	306	33.3 (0, 162.0)	168	43.5 (0, 161.3)
**Opiate Use**	*N (%) Yes*	389	124 (31.9)	198	67 (33.8)
**Visceral Disease**	*N (%) Yes*	399	33 (8.3)	199	13 (6.5)
**Treatment**					
**Treatment**	*N (%) Abiraterone*	399	323 (81.0)	199	157 (78.9)
**Concomitant Statins**	*N (%) Yes* *Atorvastatin* *Lovastatin* *Pravastatin* *Rosuvastatin* *Simvastatin* *Unknown*	0		199	199 (33.3) 107 (53.8) 3 (1.5) 11 (5.5) 33 (16.6) 22 (11.1) 23 (11.6)
**Dose of Statins**	*Median (range)*	0		122	20 (5, 80)
**Simvastatin Equivalent Dose**	*Median (range)*	0		122	30 (8, 120)
**Statins Prior to Abiraterone/Enzalutamide**	*N (%) Yes*	0		196	191 (97.5)
**Statin Use Suspended during abiraterone/enzalutamide treatment**	*N (%) Yes*	0		196	3 (1.5)
**Months, Duration of Abiraterone/Enzalutamide Treatment**	*Median (range)*	0		183	8.3 (0.4, 47.5)
**Use of a Hydrophilic Statin**	*N (%) Yes*	0		176	44 (25.0)

Characteristics of the study population, grouped by statin use.

^†^denominator is number of patients with a PSA response assessment at week 4, 8 or 12.

**Table 1D:** Outcomes of the study population, grouped by statin use

Characteristic	Statistic	*N*	No Statins	*N*	Statins
Outcomes					
**>30% PSA Decline at Week 4**	*N (%) Yes*	349	130 (37.3)	170	79 (46.5)
**>30% PSA Decline at Week 8**	*N (%) Yes*	311	136 (43.7)	169	87 (51.5)
**>30% PSA Decline at Week 12**	*N (%) Yes*	305	148 (48.5)	164	83 (50.6)
**>30% PSA Decline at 4,8 or 12 Weeks†**	*N (%) Yes*	380	186 (49.0)	194	113 (58.3)
**Overall Survival**	*N (%) Deaths* *Median (95% CI)* *6-mo OS (95% CI)* *1-year OS (95% CI)* *2-year OS (95% CI)*	399	347 (87.0) 12.9 (11.4, 14.6) 78.6 (74.2, 82.3) 53.8 (48.7, 58.7) 25.9 (21.6, 30.5)	199	166 (83.4) 20.8 (18.3, 23.2) 87.8 (82.3, 91.6) 75.0 (68.3, 80.5) 41.6 (34.5, 48.4)
**Cause of Death**	*Prostate Cancer*	347	324 (93.4)	166	144 (86.8)
**Cancer-Specific Survival**	*Median (95% CI)* *6-mo OS (95% CI)* *1-year OS (95% CI)* *2-year OS (95% CI)*	399	13.4 (12.1, 15.8) 79.3 (74.9, 83.0) 56.0 (50.9, 60.9) 27.8 (23.2, 32.5)	199	22.3 (19.2, 24.7) 89.7 (84.5, 93.2) 77.6 (71.0, 82.8) 45.5 (38.1, 52.6)
**Vascular Events**	*Cardiovascular N (%)* *Cerebrovascular N (%)* *Either N (%)*	399	8 (2.0) 3 (0.8) 11 (2.8)	199	12 (6.0) 10 (5.0) 22 (11.1)

### Statin use

Approximately one-third of the evaluable study population (199 of 598 patients) received statins during treatment, with 107 patients receiving atorvastatin (18% of patients). Importantly, statin use was documented by the local investigator using prescription data in almost 91% of cases. Only eleven patients were reported to have started statin after abiraterone or enzalutamide or to have interrupted statins before suspending abiraterone/enzalutamide treatment (2% of patients). The median simvastatin-equivalent daily dose administered was 30 mg.

### Association of statins with OS and cancer-related survival

**Figure 1:**
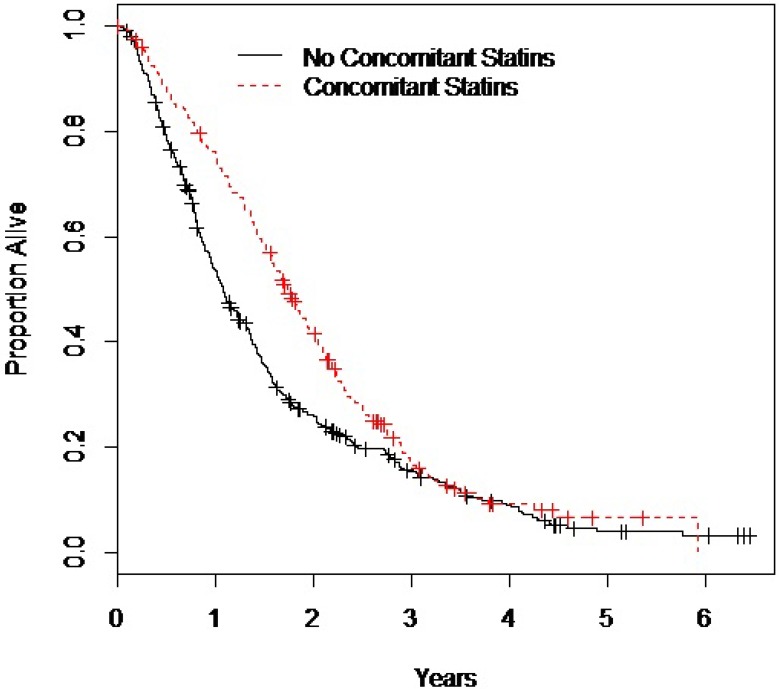
The Kaplan Meier curves for survival in patients receiving statins versus patients not receiving statins during abiraterone or enzalutamide treatment

Median OS was significantly improved for mCPRC patients who received concomitant statins, when compared to patients not treated with statins (20.8 versus 12.9 months; hazard ratio [HR] = 0.57, 95% CI = 0.46–0.71, *P* < 0.001) (Figure 1). Table 2A summarizes the results of univariable and multivariable models for OS. In the multivariable model, statin use remained strongly associated with OS with a 53% reduction in the risk of death. This association was similar in subgroup analyses and in the landmark analyses. Among the study patients who had died (*n* = 513), over 91% of the deaths were attributable to prostate cancer, and thus the cancer-specific survival was similar to OS. Median cancer-specific survival was also significantly improved for patients who received concomitant statins, when compared to patients not treated with statins (22.3 versus 13.4 months; HR = 0.43, 95% CI = 0.32 to 0.58, *P* < 0.001) (Table 2B).

**Table 2A:** Cox regression analyses, outcome = overall survival

		All Patients			Abiraterone			Enzalutamide		
	Type	*N*	HR (95% CI)	*P*	*N*	HR (95% CI)	*P*	*N*	HR (95% CI)	*P*
**Age**	*/ decade*	598	1.09 (0.98, 1.21)	0.098	480	1.06 (0.95, 1.19)	0.28	118	1.23 (0.95, 1.59)	0.12
**Months, Castrat.-Sensitive Dz**	*<12 mos vs ≥12 mos*	390	1.25 (0.97, 1.60)	0.080	300	1.15 (0.87, 1.53)	0.33	90	1.43 (0.80, 2.54)	0.23
**Months, Dx-Mets**	*<36 mos vs ≥36 mos*	474	0.94 (0.77, 1.16)	0.57	370	0.90 (0.72, 1.14)	0.40	104	1.03 (0.65, 1.65)	0.89
**Alk Phos**	*Log-transformed*	448	1.37 (1.19, 1.57)	<0.001	367	1.31 (1.13, 1.52)	<0.001	81	2.09 (1.32, 3.30)	0.002
**LDH**	*Log-transformed*	259	1.82 (1.32, 2.49)	<0.001	194	1.60 (1.12, 2.28)	0.009	65	2.15 (1.08, 4.30)	0.030
**Neutrophils/Lymphocyte Ratio**	*Log-transformed*	530	1.59 (1.36, 1.84)	<0.001	435	1.60 (1.36, 1.88)	<0.001	95	1.32 (0.85, 2.05)	0.22
**Hemoglobin**	*/ unit*	555	0.82 (0.77, 0.87)	<0.001	450	0.81 (0.75, 0.86)	<0.001	105	0.95 (0.81, 1.12)	0.52
**Baseline PSA**	*Log-transformed*	587	1.21 (1.14, 1.29)	<0.001	476	1.20 (1.13, 1.28)	<0.001	111	1.27 (1.07, 1.51)	0.007
**Charlson Score**	*/ unit≥10 vs <10*	598	1.03 (0.99, 1.08)1.10 (0.92, 1.32)	0.170.29	480	1.03 (0.98, 1.08)1.13 (0.92, 1.38)	0.280.25	118	1.03 (0.90, 1.17)0.91 (0.58, 1.43)	0.690.69
**Gleason Score**	*≥8 vs <8*	540	1.16 (0.96, 1.42)	0.13	431	1.18 (0.94, 1.48)	0.15	109	0.99 (0.63, 1.57)	0.98
**Visceral Disease**	*Yes vs No*	598	1.67 (1.18, 2.35)	0.004	480	1.59 (1.05, 2.40)	0.028	118	1.71 (0.89, 3.30)	0.11
**Opiates**	*Yes vs No*	587	0.98 (0.80, 1.21)	0.85	476	1.07 (0.86, 1.34)	0.52	111	0.52 (0.29, 0.94)	0.030
**Treatment**	*Enza vs Abi*	598	1.10 (0.87, 1.40)	0.42		-			-	
**Concomitant Statins**	*Yes vs No*	598	0.57 (0.46, 0.71)	<0.001	480	0.58 (0.45, 0.73)	<0.001	118	0.61 (0.37, 1.01)	0.052
**Statin Type**	*Atorvastatin vs Other*	199	1.05 (0.75, 1.48)	0.77	157	1.21 (0.82, 1.77)	0.34	42	0.41 (0.12, 1.38)	0.15
**Simvastatin Equivalent Dose**	*/ mg*	123	1.00 (0.99, 1.01)	0.51	100	0.99 (0.98, 1.01)	0.28	23	1.03 (0.97, 1.08)	0.33
**Use of a hydrophilic statin**	*Yes vs No*	176	0.78 (0.52, 1.16)	0.22	153	0.64 (0.42, 0.98)	0.040	23	2.29 (0.63, 8.29)	0.21
**Multivariable Model**										
**Age**	*/ decade*	387	1.10 (0.94, 1.29)	0.25	319	1.09 (0.92, 1.30)	0.33	68	1.30 (0.86, 1.98)	0.21
**Alk Pho**s	*Log-transformed*		1.24 (1.06, 1.46)	0.008		1.21 (1.02, 1.43)	0.031		2.98 (1.60, 5.58)	<0.001
**Neutrophils/Lymphocyte Ratio**	*Log-transformed*		1.56 (1.30, 1.89)	<0.001		1.63 (1.33, 2.00)	<0.001		0.93 (0.49, 1.76)	0.82
**Hemoglobin**	*/ unit*		0.85 (0.78, 0.93)	<0.001		0.83 (0.76, 0.91)	<0.001		1.04 (0.81, 1.35)	0.75
**Baseline PSA**	*Log-transformed*		1.13 (1.05, 1.22)	0.002		1.13 (1.04, 1.22)	0.003		1.35 (1.02, 1.79)	0.038
**Charlson Score**	*≥10 vs <10*		0.95 (0.72, 1.24)	0.69		0.96 (0.71, 1.30)	0.81		0.79 (0.38, 1.65)	0.53
**Gleason Score**	*≥8 vs <8*		1.13 (0.88, 1.45)	0.34		1.16 (0.88, 1.54)	0.29		1.52 (0.76, 3.03)	0.24
**Visceral Disease**	*Yes vs No*		1.93 (1.24, 3.01)	0.004		1.81 (1.08, 3.03)	0.025		3.25 (1.11, 9.53)	0.032
**Opiate Use**	*Yes vs No*		1.10 (0.85, 1.42)	0.48		1.28 (0.97, 1.68)	0.080		0.22 (0.09, 0.57)	0.002
**Treatment**	*Enza vs Abi*		0.91 (0.66, 1.25)	0.56		-	-		-	-
**Concomitant Statins**	*Yes vs No*		0.47 (0.35, 0.63)	<0.001		0.45 (0.33, 0.62)	<0.001		0.41 (0.19, 0.92)	0.031
**3-Month Landmark Analysis – Multivariable Model.**										
**Age**	*/ decade*	360	1.07 (0.91, 1.27)	0.41	296	1.06 (0.88, 1.27)	0.54	64	1.19 (0.77, 1.84)	0.43
**Alk Phos**	*Log-transformed*		1.15 (0.97, 1.36)	0.12		1.11 (0.92, 1.33)	0.27		3.13 (1.63, 6.02)	<0.001
**Neutrophils/Lymphocyte Ratio**	*Log-transformed*		1.51 (1.23, 1.85)	<0.001		1.59 (1.27, 1.97)	<0.001		0.93 (0.47, 1.83)	0.83
**Hemoglobin**	*/unit*		0.86 (0.78, 0.94)	0.001		0.85 (0.77, 0.93)	0.001		1.00 (0.75, 1.34)	0.99
**Baseline PSA**	*Log-transformed*		1.13 (1.05, 1.22)	0.002		1.13 (1.04, 1.22)	0.004		1.33 (0.97, 1.84)	0.078
**Charlson Score**	*≥10 vs <10*		0.93 (0.70, 1.22)	0.59		0.97 (0.71, 1.33)	0.86		0.75 (0.34, 1.62)	0.46
**Gleason Score**	*≥8 vs <8*		1.16 (0.89, 1.51)	0.26		1.25 (0.93, 1.69)	0.14		1.17 (0.56, 2.43)	0.67
**Visceral Disease**	*Yes vs No*		2.05 (1.28, 3.30)	0.003		1.79 (1.02, 3.13)	0.043		3.61 (1.12,10.86)	0.023
**Opiates**	*Yes vs No*		1.16 (0.89, 1.51)	0.28		1.27 (0.96, 1.69)	0.099		0.30 (0.11, 0.79)	0.015
**Treatment**	*Enza vs Abi*		0.86 (0.62, 1.20)	0.38		-	-		-	-
**≥3 Months of Continuous Use Statins**	*Yes vs No*		0.53 (0.39, 0.72)	<0.001		0.51 (0.37, 0.72)	<0.001		0.49 (0.20, 1.24)	0.13

**Table 2B:** Cox regression analyses, outcome = cancer-specific survival

		All Patients			Abiraterone Only			Enzalutamide Only		
	Type	*N*	HR (95% CI)	*P*	*N*	HR (95% CI)	*P*	*N*	HR (95% CI)	*P*
**Age**	*/ decade*	598	1.03 (0.93, 1.15)	0.60	480	1.01 (0.90, 1.13)	0.93	118	1.16 (0.88, 1.54)	0.29
**Months, Castrat.-Sensitive Dz**	*<12 mos vs ≥12 mos*	390	1.26 (0.98, 1.63)	0.073	300	1.18 (0.88, 1.57)	0.27	90	1.35 (0.74, 2.46)	0.33
**Months, Dx-Mets**	*<36 mos vs ≥36 mos*	474	0.90 (0.72, 1.11)	0.32	370	0.87 (0.69, 1.12)	0.28	104	0.97 (0.59, 1.60)	0.90
**Alk Phos**	*Log-transformed*	448	1.38 (1.19, 1.59)	<0.001	367	1.32 (1.13, 1.54)	<0.001	81	2.34 (1.44, 3.80)	<0.001
**LDH**	*Log-transformed*	259	1.94 (1.41, 2.69)	<0.001	194	1.68 (1.17, 2.42)	0.005	65	2.50 (1.24, 5.03)	0.010
**Neutrophils/Lymphocyte Ratio**	*Log-transformed*	530	1.70 (1.45, 1.99)	<0.001	435	1.70 (1.43, 2.02)	<0.001	95	1.37 (0.85, 2.21)	0.20
**Hemoglobin**	*/ unit*	555	0.80 (0.75, 0.86)	<0.001	450	0.79 (0.74, 0.85)	<0.001	105	0.92 (0.78, 1.10)	0.36
**Baseline PSA**	*Log-transformed*	587	1.23 (1.16, 1.31)	<0.001	476	1.22 (1.14, 1.30)	<0.001	111	1.29 (1.07, 1.56)	0.008
**Charlson Score**	*/ unit≥10 vs <10*	598	1.00 (0.96, 1.06)1.02 (0.84, 1.22)	0.880.88	480	1.00 (0.94, 1.05)1.04 (0.84, 1.27)	0.890.74	118	1.01 (0.88, 1.15)0.79 (0.48, 1.29)	0.940.34
**Gleason Score**	*≥8 vs <8*	540	1.21 (0.98, 1.48)	0.077	431	1.20 (0.95, 1.52)	0.13	109	1.05 (0.64, 1.72)	0.85
**Visceral Disease**	*Yes vs No*	598	1.66 (1.16, 2.36)	0.005	480	1.52 (0.99, 2.33)	0.056	118	1.86 (0.95, 3.62)	0.069
**Opiates**	*Yes vs No*	587	1.01 (0.82, 1.26)	0.91	476	1.08 (0.86, 1.36)	0.51	111	0.59 (0.32, 1.09)	0.094
**Treatment**	*Enza vs Abi*	598	1.04 (0.81, 1.34)	0.77		-			-	
**Concomitant Statins**	*Yes vs No*	598	0.51 (0.41, 0.64)	<0.001	480	0.52 (0.40, 0.67)	<0.001	118	0.53 (0.31, 0.93)	0.025
**Statin Type**	*Atorvastatin vs Other*	199	1.04 (0.72, 1.51)	0.84	157	1.17 (0.78, 1.78)	0.45	42	0.41 (0.12, 1.38)	0.15
**Simvastatin Equivalent Dose**	*/ mg*	123	1.00 (0.99, 1.01)	0.47	100	0.99 (0.98, 1.01)	0.26	23	1.03 (0.97, 1.08)	0.33
**Use of a hydrophilic statin**	*Yes vs No*	176	0.77 (0.50, 1.18)	0.24	153	0.62 (0.39, 0.98)	0.043	23	2.29 (0.63, 8.29)	0.21
**Multivariable Model**										
**Age**	*/ decade*	387	1.03 (0.87, 1.21)	0.77	319	1.03 (0.86, 1.23)	0.79	68	1.25 (0.79, 1.99)	0.34
**Alk Phos**	*Log-transformed*		1.21 (1.02, 1.43)	0.026		1.18 (0.98, 1.40)	0.075		3.91 (1.88, 8.10)	<0.001
**Neutrophils/Lymphocyte Ratio**	*Log-transformed*		1.62 (1.33, 1.98)	<0.001		1.66 (1.35, 2.06)	<0.001		0.98 (0.48, 2.00)	0.95
**Hemoglobin**	*/ unit*		0.84 (0.77, 0.92)	<0.001		0.83 (0.75, 0.91)	<0.001		0.99 (0.76, 1.30)	0.97
**Baseline PSA**	*Log-transformed*		1.16 (1.07, 1.26)	<0.001		1.15 (1.06, 1.25)	<0.001		1.44 (1.05, 1.98)	0.024
**Charlson Score**	*≥10 vs <10*		0.93 (0.70, 1.23)	0.61		0.95 (0.70, 1.30)	0.74		0.63 (0.29, 1.38)	0.25
**Gleason Score**	*≥8 vs <8*		1.13 (0.87, 1.47)	0.35		1.15 (0.85, 1.54)	0.36		1.76 (0.82, 3.78)	0.15
**Visceral Disease**	*Yes vs No*		1.87 (1.17, 2.97)	0.008		1.68 (0.97, 2.90)	0.063		4.17 (1.34,12.96)	0.013
**Opiates**	*Yes vs No*		1.15 (0.88, 1.51)	0.31		1.29 (0.97, 1.72)	0.080		0.19 (0.07, 0.52)	0.001
**Treatment**	*Enza vs Abi*		0.87 (0.62, 1.22)	0.41		-	-		-	-
**Concomitant Statins**	*Yes vs No*		0.43 (0.32, 0.58)	<0.001		0.41 (0.29, 0.57)	<0.001		0.37 (0.16, 0.87)	0.023
**3-Month Landmark Analysis – Multivariable Model**										
**Age**	*/ decade*	360	0.98 (0.83, 1.17)	0.86	296	0.98 (0.81, 1.19)	0.87	64	1.05 (0.66, 1.70)	0.83
**Alk Phos**	*Log-transformed*		1.10 (0.92, 1.32)	0.29		1.06 (0.88, 1.29)	0.52		4.00 (1.87, 8.58)	<0.001
**Neutrophils/Lymphocyte Ratio**	*Log-transformed*		1.56 (1.26, 1.94)	<0.001		1.62 (1.28, 2.03)	<0.001		0.97 (0.46, 2.06)	0.94
**Hemoglobin**	*/ unit*		0.85 (0.77, 0.94)	0.001		0.84 (0.76, 0.93)	0.001		0.98 (0.73, 1.33)	0.91
**Baseline PSA**	*Log-transformed*		1.16 (1.07, 1.26)	<0.001		1.15 (1.06, 1.26)	0.001		1.34 (0.95, 1.90)	0.096
**Charlson Score**	*≥10 vs <10*		0.92 (0.69, 1.23)	0.56		0.96 (0.69, 1.32)	0.78		0.64 (0.28, 1.45)	0.28
**Gleason Score**	*≥8 vs <8*		1.17 (0.89, 1.54)	0.27		1.25 (0.91, 1.71)	0.17		1.17 (0.53, 2.58)	0.70
**Visceral Disease**	*Yes vs No*		1.97 (1.19, 3.23)	0.008		1.64 (0.90, 3.00)	0.10		4.21 (1.36,13.07)	0.013
**Opiates**	*Yes vs No*		1.20 (0.91, 1.58)	0.21		1.28 (0.95, 1.72)	0.10		0.27 (0.10, 0.77)	0.015
**Treatment**	*Enza vs Abi*		0.84 (0.59, 1.20)	0.33		-	-		-	-
**≥3 Months of Continuous Use Statins**	*Yes vs No*		0.48 (0.35, 0.66)	<0.001		0.47 (0.33, 0.66)	<0.001		0.46 (0.17, 1.22)	0.12

No statistically significant treatment effects were observed between enzalutamide versus abiraterone, nor were treatment differences observed based on type (atorvastatin versus other) or dose of statin.

### Association of statins with PSA response

Among the 574 patients with available information, 299 (52.1%) experienced a PSA response (> 30% decline) within 12 weeks of abiraterone or enzalutamide initiation. Early PSA responses were observed significantly more often in patients that received statins, when compared to patients who did not receive statin therapy ( 58% versus 49%; odds ratio [OR] = 1.46, 95% CI = 1.02–2.08, *P* = 0.04) (Table 3). The association between early PSA response and statin use remained significant in the multivariable analysis (OR = 1.63, 95% CI = 1.03–2.60, *P* = 0.04).

**Table 3:** Logistic regression analyses, outcome = early 30% PSA decline

		All Patients			Abiraterone			Enzalutamide		
	Type	*N*	OR (95% CI)	*P*	*N*	OR (95% CI)	*P*	*N*	OR (95% CI)	*P*
**Age**	*/decade*	574	1.05 (0.87, 1.27)	0.63	465	1.20 (0.96, 1.48)	0.10	109	0.60 (0.35, 1.02)	0.059
**Months, Castration-sensitive Disease**	*<12 mos vs ≥12 mos*	376	0.77 (0.49, 1.22)	0.27	293	0.69 (0.41, 1.17)	0.17	83	1.07 (0.40, 2.92)	0.89
**Months, Disease-Metastases**	*<36 mos vs ≥36 mos*	457	0.72 (0.49, 1.05)	0.085	360	0.62 (0.40, 0.96)	0.031	97	1.09 (0.46, 2.54)	0.85
**Alk Phos**	*Log-transformed*	433	1.10 (0.85, 1.41)	0.49	355	1.18 (0.89, 1.56)	0.26	78	0.74 (0.39, 1.37)	0.33
**LDH**	*Log-transformed*	255	0.72 (0.41, 1.26)	0.24	192	0.91 (0.47, 1.75)	0.78	63	0.44 (0.14, 1.42)	0.17
**Neutrophils/Lymphocyte Ratio**	*Log-transformed*	516	0.99 (0.76, 1.28)	0.91	423	0.97 (0.73, 1.30)	0.86	93	1.46 (0.68, 3.15)	0.34
**Hemoglobin**	*/unit*	540	1.17 (1.04, 1.32)	0.008	438	1.16 (1.01, 1.32)	0.034	102	1.15 (0.90, 1.46)	0.27
**Baseline PSA**	*Log-transformed*	572	1.02 (0.91, 1.14)	0.75	464	1.03 (0.92, 1.17)	0.60	108	1.00 (0.76, 1.32)	0.99
**Charlson Score**	*/unit≥10 vs <10*	574	1.02 (0.94, 1.12)1.07 (0.76, 1.50)	0.620.71	465	1.06 (0.96, 1.17)1.36 (0.93, 1.99)	0.230.11	109	0.86 (0.67, 1.10)0.43 (0.19, 1.00)	0.220.051
**Gleason Score**	*≥8 vs <8*	520	0.58 (0.40, 0.85)	0.005	419	0.54 (0.35, 0.83)	0.005	101	0.88 (0.39, 2.01)	0.76
**Visceral Disease**	*Yes vs No*	574	0.52 (0.27, 1.00)	0.050	465	0.71 (0.33, 1.52)	0.38	109	0.32 (0.09, 1.09)	0.068
**Opiate Use**	*Yes vs No*	571	0.92 (0.62, 1.37)	0.69	463	0.93 (0.60, 1.44)	0.74	108	1.04 (0.38, 2.82)	0.94
**Treatment**	*Enzalutamide vs Abiraterone*	574	0.95 (0.61, 1.47)	0.81		-			-	
**Concomitant Statins**	*Yes vs No*	574	1.46 (1.02, 2.08)	0.040	465	1.57 (1.05, 2.34)	0.030	109	1.09 (0.48, 2.48)	0.85
**Statin Type**	*Atorvastatin vs Other*	194	0.76 (0.40, 1.42)	0.38	154	0.77 (0.38, 1.58)	0.48	40	0.59 (0.10, 3.59)	0.56
**Dose of Statins**	*/mg*	122	1.00 (0.98, 1.01)	0.60	99	1.00 (0.98, 1.02)	0.93	23	0.92 (0.84, 1.02)	0.11
**Use of a hydrophilic statin**	*Yes vs No*	173	1.06 (0.52, 2.16)	0.88	150	1.18 (0.55, 2.55)	0.67	23	0.76 (0.10, 5.94)	0.80
**Multivariable Model**										
**Age**	*/decade*	379	0.98 (0.72, 1.33)	0.87	312	1.04 (0.74, 1.47)	0.83	67	0.76 (0.35, 1.64)	0.48
**Alk Phos**	*Log-transformed*		1.06 (0.78, 1.44)	0.70		1.17 (0.84, 1.64)	0.36		0.51 (0.20, 1.31)	0.16
**Neutrophils/Lymphocyte Ratio**	*Log-transformed*		1.16 (0.83, 1.62)	0.37		1.15 (0.80, 1.65)	0.46		1.93 (0.62, 6.01)	0.26
**Hemoglobin**	*/unit*		1.22 (1.04, 1.44)	0.015		1.21 (1.01, 1.45)	0.043		1.11 (0.76, 1.62)	0.60
**Baseline PSA**	*Log-transformed*		1.09 (0.93, 1.26)	0.29		1.06 (0.90, 1.25)	0.50		1.26 (0.78, 2.02)	0.35
**Charlson Score**	*≥10 vs <10*		1.07 (0.65, 1.77)	0.80		1.31 (0.74, 2.31)	0.36		0.40 (0.11, 1.42)	0.16
**Gleason Score**	*≥8 vs <8*		0.69 (0.43, 1.10)	0.12		0.73 (0.43, 1.23)	0.24		0.80 (0.26, 2.50)	0.70
**Visceral Disease**	*Yes vs No*		0.66 (0.28, 1.53)	0.33		0.74 (0.26, 2.08)	0.57		0.51 (0.11, 2.41)	0.40
**Opiates**	*Yes vs No*		0.97 (0.59, 1.57)	0.89		1.09 (0.64, 1.87)	0.75		0.77 (0.19, 3.20)	0.72
**Treatment**	*Enza vs Abi*		1.45 (0.81, 2.60)	0.21		-	-		-	-
**Concomitant Statins**	*Yes vs No*		1.63 (1.03, 2.60)	0.039		1.80 (1.06, 3.06)	0.029		1.02 (0.32, 3.21)	0.97

### Association of statin use and cardiovascular or cerebrovascular events

Thirty-three study patients experienced a cardiovascular or cerebrovascular event during the time period analyzed. Timing of events was not consistently reported, and therefore time-to-event analyses could not be performed. Among the 199 patients prescribed statins, 12 (6.0%) experienced a cardiovascular event, and 10 (5.0%) experienced a cerebrovascular event. In contrast, among the 399 patients not prescribed statin therapy, 8 (2.0%) experienced a cardiovascular event, and 3 (0.8%) experienced a cerebrovascular event. After adjusting for other factors in a multivariable model, concomitant statin use remained a significant predictive factor of increased risk of cardiovascular or cerebrovascular events (OR = 3.24, 95% CI = 1.15–9.17, *p*-value = 0.03) (Table 4).

**Table 4:** Logistic regression analyses of cardiovascular or cerebrovascular events

		All Patients		
	Type	*N*	OR (95% CI)	*P*
**Age**	*/decade*	598	2.24 (1.46, 3.46)	<0.001
**Months, Castration-sensitive Disease**	*<12 mos vs ≥12 mos*	390	0.55 (0.16, 1.97)	0.36
**Months, Disease-Metastases**	*<36 mos vs ≥36 mos*	474	1.14 (0.51, 2.55)	0.75
**Alk Phos**	*Log-transformed*	448	0.94 (0.54, 1.64)	0.83
**LDH**	*Log-transformed*	259	0.89 (0.26, 3.03)	0.85
**Neutrophils/Lymphocyte Ratio**	*Log-transformed*	530	1.38 (0.81, 2.36)	0.24
**Hemoglobin**	*/unit*	555	1.02 (0.80, 1.31)	0.85
**PSA at Diagnosis**	*Log-transformed*	587	0.94 (0.74, 1.18)	0.57
**Charlson Score**	*/unit* *≥10 vs <10*	598	1.54 (1.29, 1.84) 4.51 (1.72,11.85)	<0.001 0.002
**Gleason Score**	*≥8 vs <8*	540	0.57 (0.27, 1.19)	0.13
**Visceral Disease**	*Yes vs No*	598	0.36 (0.05, 2.71)	0.32
**Opiate Use**	*Yes vs No*	587	0.68 (0.30, 1.54)	0.35
**Treatment**	*Enzalutamide vs Abiraterone*	598	0.90 (0.36, 2.23)	0.82
**Concomitant Statins**	*Yes vs No*	598	4.38 (2.08, 9.24)	<0.001
**Statin Type**	*Atorvastatin vs Other*	199	1.58 (0.63, 3.96)	0.33
**Dose of Statins**	*/mg*	123	1.02 (0.99, 1.04)	0.22
**Use of a hydrophilic statin**	*Yes vs No*	176	0.73 (0.23, 2.30)	0.58
**Multivariable Analysis**				
**Age**	*/decade*	387	2.56 (1.11, 5.89)	0.028
**Alk Phos**	*Log-transformed*		1.39 (0.61, 3.19)	0.43
**Neutrophils/Lymphocyte Ratio**	*Log-transformed*		1.24 (0.52, 2.94)	0.63
**Hemoglobin**	*/unit*		1.11 (0.73, 1.70)	0.62
**PSA at Diagnosis**	*Log-transformed*		0.65 (0.45, 0.93)	0.020
**Charlson Score**	*≥10 vs <10*		1.56 (0.43, 5.70)	0.50
**Gleason Score**	*≥8 vs <8*		0.77 (0.24, 2.46)	0.66
**Visceral Disease**	*Yes vs No*		0.64 (0.07, 6.28)	0.70
**OpiateUse**	*Yes vs No*		0.72 (0.22, 2.38)	0.59
**Treatment**	*Enzalutamide vs Abiraterone*		0.58 (0.12, 2.78)	0.50
**Concomitant Statins**	*Yes vs No*		3.24 (1.15, 9.17)	0.027

## DISCUSSION

Although statin use has been associated with reduced cancer-related mortality in a variety of malignancies [13], the potential synergism of statins with anti-cancer medications has been prospectively investigated only in a few clinical trials. Data from the recently published phase III double-blind, placebo-controlled LUNGSTAR trial failed to detect an OS or progression-free survival (PFS) benefit when pravastatin was added to first-line standard chemotherapy in patients with small-cell lung cancer [14]. Similarly, no benefit in overall survival associated with the use of statins added to chemotherapy was reported in two additional phase III trials conducted in advanced gastric [15] and colorectal [16] cancer patients, respectively.

Biologically, statins can potentiate the efficacy of anti-androgen treatments, such as abiraterone and enzalutamide, in mCRPC through a number of potential mechanisms, including: inhibition of intra-tumoral *de novo* steroid biosynthesis [17], inhibition of biosynthesis of isoprenoids [18], as well as inhibition of the organic anionic transporters (e.g., SLCO2B1) [19] that are responsible for adrenal androgen dehydroepiandrosterone (DHEA) influx into cancer cells [20].

In one translational study, Harshman *et al.* [21] showed that statins impaired DHEA influx through competitive inhibition of the SLCO2B1 transporter both in both androgen-dependent (LNCaP) and partially androgen-dependent (22RV1) prostate cancer cell lines. This was supported by their retrospective clinical study of 926 patients, treated with androgen deprivation, which demonstrated that patients who received statin therapy experienced longer median time to progression, when compared to patients not treated with a statin (27.5 versus 17.4 months; *P* < 0.001). Because abiraterone is also a SLCO2B1 substrate, the same research group [10] hypothesized that statin use could be a negative predictive factor for patients taking abiraterone. However, their retrospective study of 224 abiraterone-treated patients demonstrated that statin use trended toward longer treatment duration (14.2 versus 9.2 months; HR: 0.79, 95% CI, 0.57–1.09, *P* = 0.14). Despite lack of validation in an independent cohort of 270 abiraterone-treated patients [10], the authors concluded that concomitant stain use did not negatively impact survival.

In our previous retrospective observational study (*n* = 187 mCRPC patients from 10 participating centers who received abiraterone), statin use was associated with longer OS in univariate (HR = 0.51, 95% CI = 0.37–0.72, *P* < 0.001) and multivariate analyses (HR = 0.40, 95% CI = 0.27–0.59, *P* < 0.001). Statin use was also significantly associated with early PSA declines (>50% declines at week 12 in statin users versus non-users: 72.1% vs. 38.5; *P* < 0.001). This study was limited by several factors, including the relatively small sample size, the lack of information about statin type and statin treatment duration, comorbidities, cardiovascular events, and prostate cancer–specific survival. To overcome these limitations, we designed a retrospective observational study to be conducted in an international setting that could better define concomitant treatment with statins. One of the purposes of the STABEN trial was to assess whether the potential advantage associated with statin use could be related to their known cardiovascular and cerebrovascular protective effect, of particular potential importance in an elderly population receiving abiraterone – an agent with known cardiovascular toxicity [22]. In the present retrospective study, multivariable models that included known prognostic factors in prostate cancer (e.g., baseline PSA levels, hemoglobin levels, Gleason score, alkaline phosphatase and LDH levels [23], visceral involvement [24] and neutrophil to lymphocyte ratio [25]) revealed that statin use was associated with a 53% reduction in the risk of all-cause mortality, and a 57% reduction in the risk of prostate cancer-specific mortality. It also appeared that statin co-administration increased the odds of having an early >30% PSA decrease, which is consistent with our previously reported findings and adds strength to the hypothesis of a potential synergism with abiraterone/enzalutamide.

Notably, the positive effect of statins on survival did not appear to be influenced by the known protective statin effect against vascular events. While the observed incidence of cardiovascular events reported in this study are consistent with previously reported rates of grade 2 or higher abiraterone-associated cardiovascular events [22], mCRPC patients from this study who were prescribed statin therapy appeared to be at an approximately 4-fold greater risk of experiencing a vascular event. Although such analyses did not account for time-to-event, competing risks, or a history of pre-existing cardiac conditions, this finding could be explained by the observation that patients prescribed statins often present with a greater number of co-morbidities and therefore a greater cardio- and cerebro-vascular risk, when compared to non-statin users [26]. Furthermore, the protective effect of statins was maintained after correcting for Charlson comorbidity index. Although statin consumption was modeled by using a binary variable, it must be noted that only a few patients were not prescribed statins throughout the entire abiraterone or enzalutamide treatment duration, which does not make useful to model statin exposure as a time-dependent variable. Finally, the novelty of the STABEN study also relies in the increased survival in mCRPC patients receiving concomitant enzalutamide and statins vs. enzalutamide alone, which is consistent with the multiple putative pharmacodynamic interactions of statins with anti-androgen receptor agents.

Despite its larger sample size versus published series [8–10], this study still suffers from the limitations that apply to retrospective studies, including the lack of data on some key factors such as LDH and time from castration-sensitive disease, as well as the non-systematic selection of participating centers.

## CONCLUSIONS

In the large retrospective, observational STABEN study, we found a positive association of statin use with overall- and cancer specific- survival in patients receiving abiraterone or enzalutamide in the second-line setting after docetaxel failure. Statin use was documented by using high-quality prescription data in most patients. The positive association found in our patient cohort with survival was reported both in abiraterone- and enzalutamide-treated men and was consistent with early >30% PSA declines. Analyzed together with previous epidemiology and biological findings, the STABEN results may serve as the basis to design prospective clinical trials assessing the value of adding statins to abiraterone or enzalutamide in mCRPC patients. Optimizing statin use in patients with advanced prostate cancer represents a compelling clinical opportunity to improve survival via the addition of a safe and inexpensive drug.

## MATERIALS AND METHODS

### Inclusion criteria

Medical records were reviewed at eight participating centers for patients with diagnosed mCRPC who were treated with second-line abiraterone or enzalutamide between January 2011 and January 2016. Histologically-confirmed prostate cancer and previous docetaxel-based treatment were required for inclusion in this study. Castration-resistance was determined per Prostate Cancer Clinical Trials Working Group 2 (PCWG2) criteria [22]. Patients who received at least one 28-day cycle of abiraterone or enzalutamide in the second-line setting were regarded as eligible for this study. Patient data including medical and prostate cancer history, demographic, and baseline characteristics were retrieved starting at the time of abiraterone or enzalutamide initiation. Data collected regarding statin use included: type and dose of statin prescribed, source of the data (claims versus prescription data), and dates of statin use initiation and discontinuation.

### Data analysis

Summary statistics were used to describe patient outcomes. Time-to-event outcomes were calculated from the first date of treatment with abiraterone or enzalutamide.

The primary objective of this study was to determine whether concomitant statin therapy was predictive of OS improvement for mCRPC patients treated with second-line abiraterone or enzalutamide. The secondary objective of the study was to determine whether concomitant statin therapy was predictive of early (≤ 12 weeks) >30% PSA declines. The Kaplan-Meier method was used to estimate differences in survival between mCRPC patients treated who did and did not receive statin therapy, while Cox proportional hazards regression was used to investigate prognostic factors of overall survival. Logistic regression was used to investigate predictive factors of early >30% PSA declines. Using Cox proportional hazards, multivariable models were constructed to examine the effects of concomitant statins after adjusting for all other potential sources of variation. However, there were large numbers of missing data for some factors. Thus, *a priori*, it was decided to include only those factors which had <30% missing data and were significant on univariate analysis, or those factors with <15% missing data overall. The impact of statins was then assessed after adjusting for factors included in the multivariable model. Supportive analyses were performed by including only those treated with abiraterone (∼80% of the cohort), only those treated with enzalutamide, by performing a cancer-specific survival analysis and by performing a landmark analysis using 3-months as the landmark time. For the purposes of the landmark analysis, any patient who was not prescribed statin therapy at the time of abiraterone or enzalutamide initiation, experienced interruption of statin therapy, or received less than 3 months of statin therapy, was deemed to not have received statins. Data modifications were performed for statistical purposes. Specifically, a logarithmic transformation was used on covariates which were highly non-normal. Duration from prostate cancer diagnosis to detection of metastases, and duration of prostate cancer diagnosis to determination of castration-resistant disease were dichotomized. All analyses included site as a stratification factor. All tests were two-sided and a *p*-value of 0.05 or less was considered statistically significant. No *p*-value adjustments were performed due to multiple testing; however, inferences were performed understanding that multiple analyses were performed.
